# P327: A situational analysis of pharmacovigilance in republic of benin based on the Artemisinin-based Combination Therapies (ACTs)

**DOI:** 10.1186/2047-2994-2-S1-P327

**Published:** 2013-06-20

**Authors:** A Aurel Constant, N Jude, M Achille, H Yves

**Affiliations:** 1 Unité de Pharmacologie, Faculté des Sciences de la Santé, Université d’Abomey-Calavi, Abomey, Benin; 2 Management Sciences for Health, Strengthening Pharmaceutical Systems (SPS) Programs, Arlington, Virgin Islands, USA; 3 Unité d’enseignement et de recherche en Parasitologie, Faculté des Sciences de la Santé de Cotonou, Université d’Abomey-Calavi, Abomey, Benin; 4 Unité de Pharmacologie clinique, Cliniques Universitaires Saint-Luc, Bruxelles, Belgium

## Introduction

To assess Benin’s pharmacovigilance system, identify the gaps and define strategy which could lead to its functional establishment, structure questionnaires was applied to investigate physicians, pharmacists and pharmaceutical industry representatives’ knowledge, attitude and practice regarding Adverse Drug Reactions (ADRs) reporting.

## Methods

Indicator-based Pharmacovigilance Assessment Tool (IPAT) was also used to assess the current landscape with different stakeholders.

## Results

30.77% physicians and 31.11% pharmacists acknowledged that they faced at least one time ADRs suspected to be associated with antimalarial drug (P-value<0.01). However none of the physicians or the pharmacists has ever reported ADRs to the national pharmacovigilance service. The main reasons for not reporting were ‘‘yellow card not available’’ and “not aware about the existence of pharmacovigilance center”. 6.97 % of representatives of the pharmaceutical companies monitored the safety of their products and none of them have ever reported ADRs to the health authority (DPM). In return, none of the laboratories have ever received a report related to quality or ADRs related to their drugs on the market from DPM.

IPAT led to low overall scores indicating that there is no functional pharmacovigilance system in place. The major shortcoming is the lack of expertise in pharmacovigilance despite the availability of qualified human resource in the country.

## Conclusion

There is a need to identify and implement adequate human resources use in order to build capacity and sustain the drug safety system for essential medicines. Faculty of Medicine and researchers should be involved for the successful implementation of pharmacovigilance in Benin**.**

## Disclosure of interest

None declared

